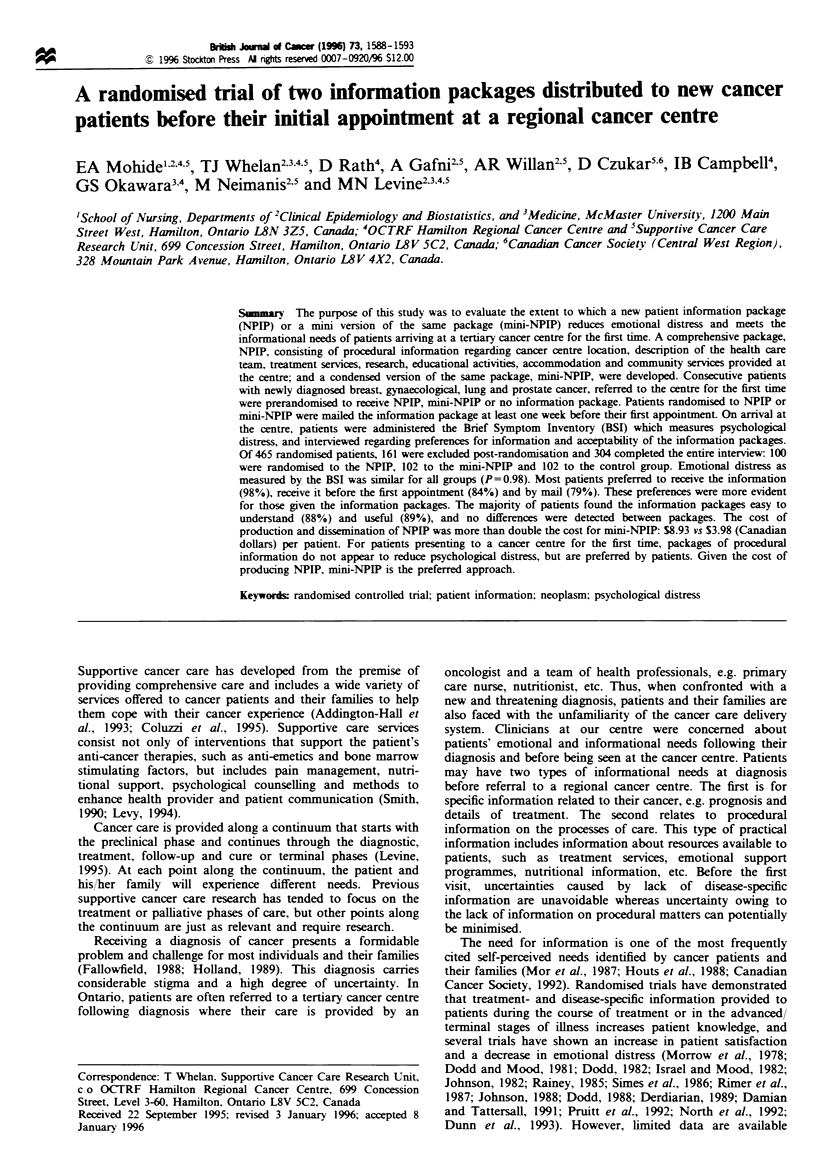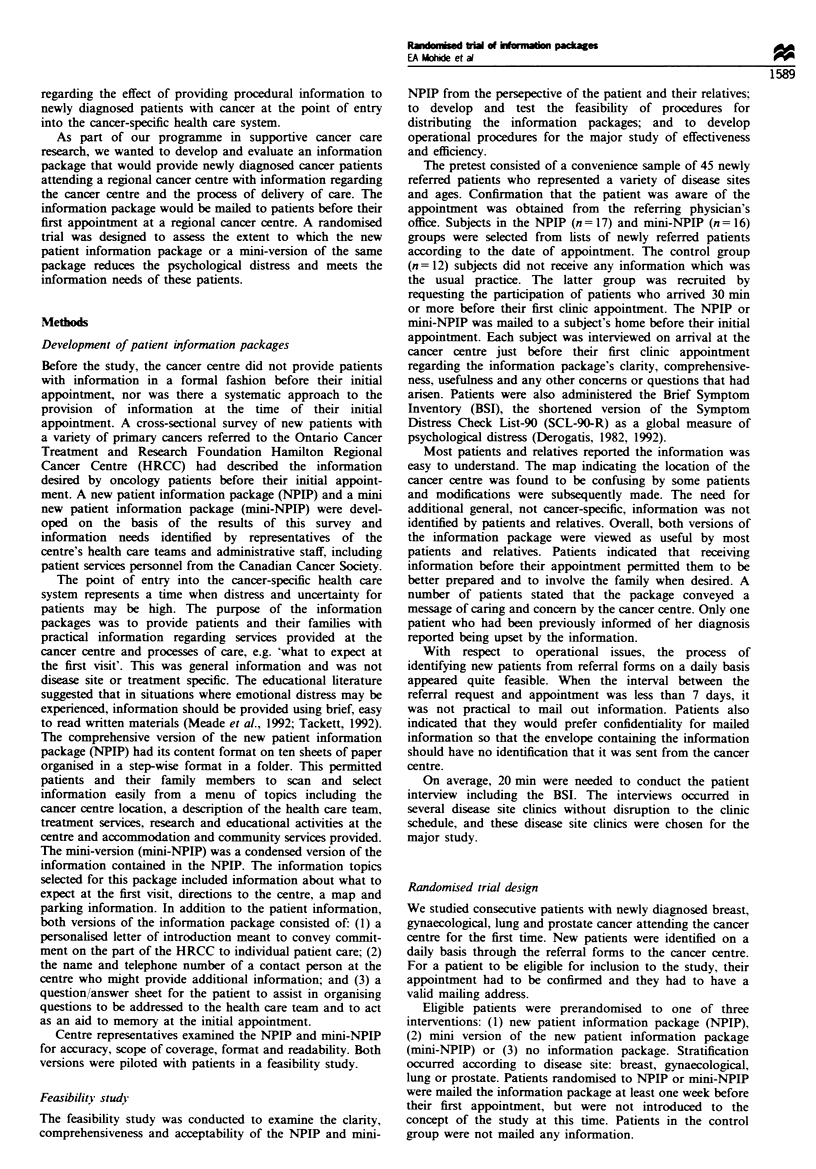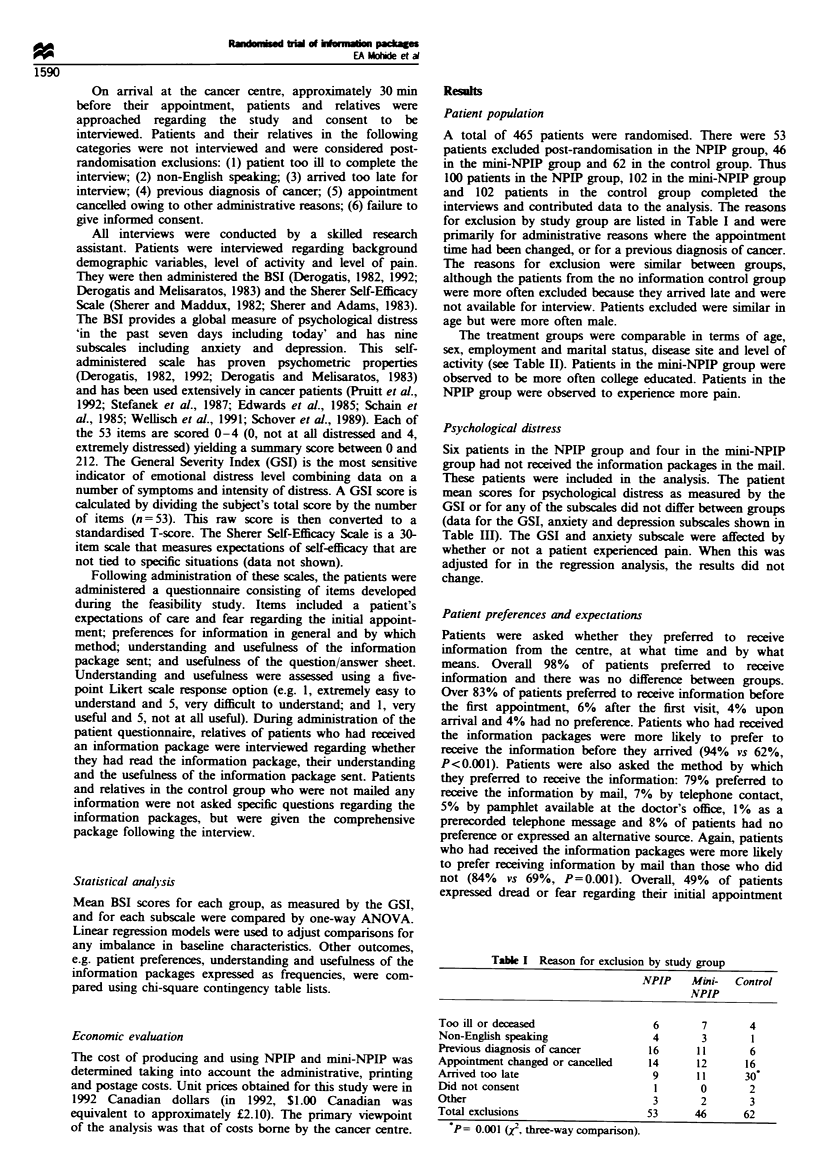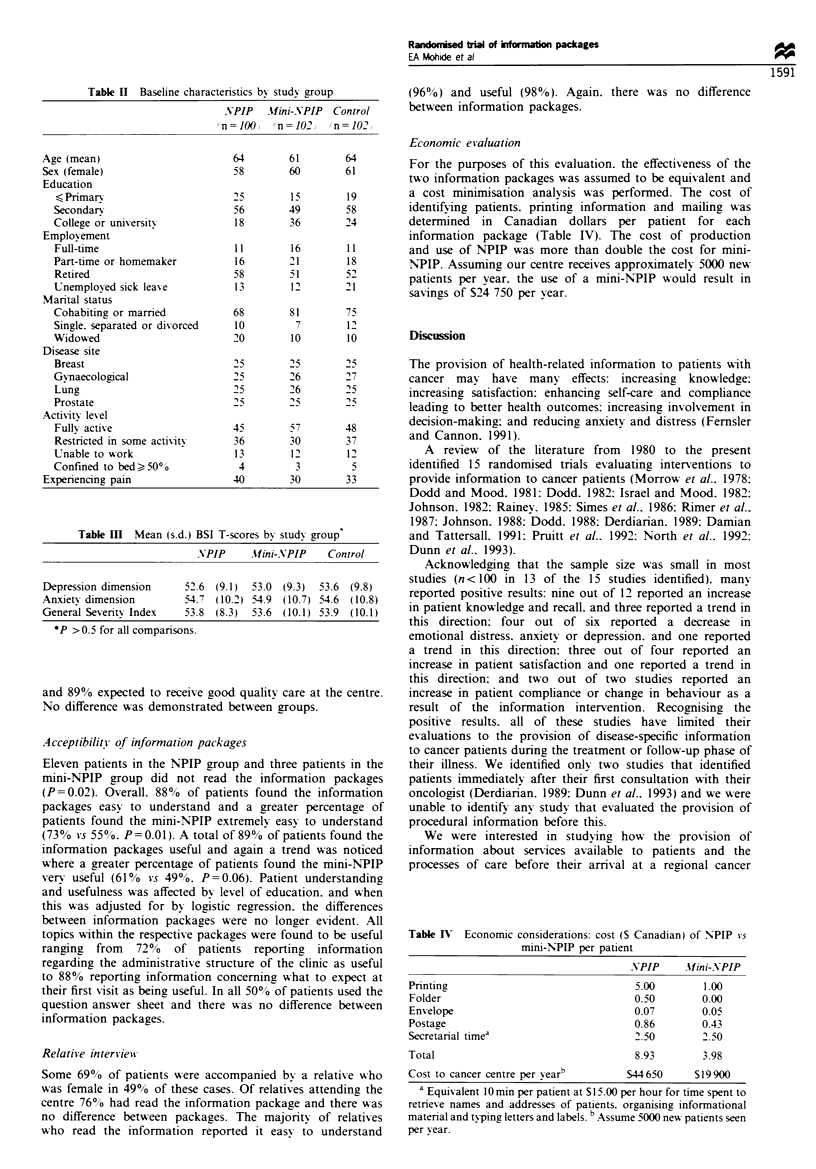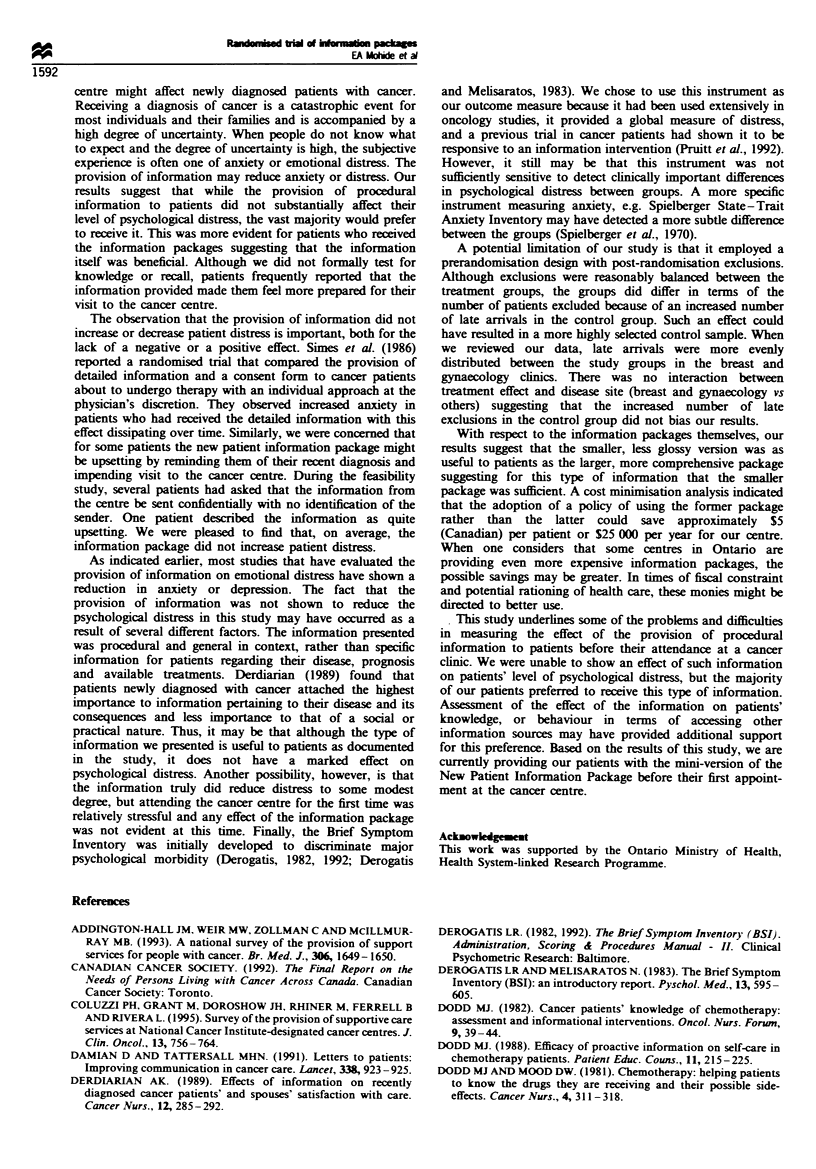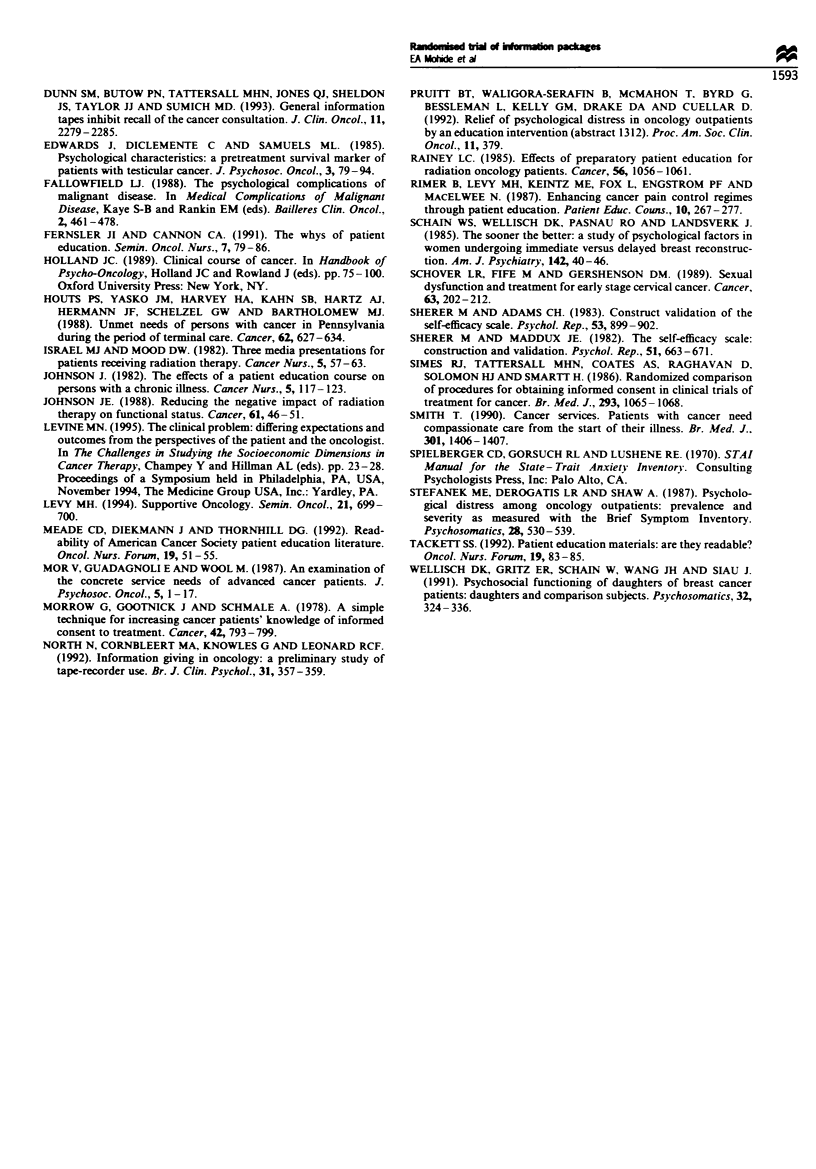# A randomised trial of two information packages distributed to new cancer patients before their initial appointment at a regional cancer centre.

**DOI:** 10.1038/bjc.1996.299

**Published:** 1996-06

**Authors:** E. A. Mohide, T. J. Whelan, D. Rath, A. Gafni, A. R. Willan, D. Czukar, I. B. Campbell, G. S. Okawara, M. Neimanis, M. N. Levine

**Affiliations:** School of Nursing, Department of Clinical Epidemiology and Biostatistics, McMaster University, Hamilton, Ontario, Canada.

## Abstract

The purpose of this study was to evaluate the extent to which a new patient information package (NPIP) or a mini version of the same package (mini-NPIP) reduces emotional distress and meets the informational needs of patients arriving at a tertiary cancer centre for the first time. A comprehensive package, NPIP, consisting of procedural information regarding cancer centre location, description of the health care team, treatment services, research, educational activities, accommodation and community services provided at the centre; and a condensed version of the same package, mini-NPIP, were developed. Consecutive patients with newly diagnosed breast, gynaecological, lung and prostate cancer, referred to the centre for the first time were prerandomised to receive NPIP, mini-NPIP or no information package. Patients randomised to NPIP or mini-NPIP were mailed the information package at least one week before their first appointment. On arrival at the centre, patients were administered the Brief Symptom Inventory (BSI) which measures psychological distress, and interviewed regarding preferences for information and acceptability of the information packages. Of 465 randomised patients, 161 were excluded post-randomisation and 304 completed the entire interview: 100 were randomised to the NPIP, 102 to the mini-NPIP and 102 to the control group. Emotional distress as measured by the BSI was similar for all groups (P = 0.98). Most patients preferred to receive the information (98%), receive it before the first appointment (84%) and by mail (79%). These preferences were more evident for those given the information packages. The majority of patients found the information packages easy to understand (88%) and useful (89%), and no differences were detected between packages. The cost of production and dissemination of NPIP was more than double the cost for mini-NPIP: $ 8.93 vs $ 3.98 (Canadian dollars) per patient. For patients presenting to a cancer centre for the first time, packages of procedural information do not appear to reduce psychological distress, but are preferred by patients. Given the cost of producing NPIP, mini-NPIP is the preferred approach.


British Journal of Cancer (1996) 73, 1588–1593

© 1996 Stockton Press All rights reserved 0007–0920/96 $12.00

### A randomised trial of two information packages distributed to new cancer patients before their initial appointment at a regional cancer centre

EA Mohide[1,2,4,5], TJ Whelan[2,3,4,5], D Rath[4], A Gafni[2,5], AR Willan[2,5], D Czukar[5,6], IB Campbell[4], GS Okawara[3,4], M Neimanis[2,5] and MN Levine[2,3,4,5]

*[1]School of Nursing, Departments of [2]Clinical Epidemiology and Biostatistics, and [3]Medicine, McMaster University, 1200 Main Street West, Hamilton, Ontario L8N 3Z5, Canada; [4]OCTRF Hamilton Regional Cancer Centre and [5]Supportive Cancer Care Research Unit, 699 Concession Street, Hamilton, Ontario L8V 5C2, Canada; [6]Canadian Cancer Society (Central West Region), 328 Mountain Park Avenue, Hamilton, Ontario L8V 4X2, Canada.*

**Summary** The purpose of this study was to evaluate the extent to which a new patient information package (NPIP) or a mini version of the same package (mini-NPIP) reduces emotional distress and meets the informational needs of patients arriving at a tertiary cancer centre for the first time. A comprehensive package, NPIP, consisting of procedural information regarding cancer centre location, description of the health care team, treatment services, research, educational activities, accommodation and community services provided at the centre; and a condensed version of the same package, mini-NPIP, were developed. Consecutive patients with newly diagnosed breast, gynaecological, lung and prostate cancer, referred to the centre for the first time were prerandomised to receive NPIP, mini-NPIP or no information package. Patients randomised to NPIP or mini-NPIP were mailed the information package at least one week before their first appointment. On arrival at the centre, patients were administered the Brief Symptom Inventory (BSI) which measures psychological distress, and interviewed regarding preferences for information and acceptability of the information packages. Of 465 randomised patients, 161 were excluded post-randomisation and 304 completed the entire interview: 100 were randomised to the NPIP, 102 to the mini-NPIP and 102 to the control group. Emotional distress as measured by the BSI was similar for all groups ($P=0.98$). Most patients preferred to receive the information (98%), receive it before the first appointment (84%) and by mail (79%). These preferences were more evident for those given the information packages. The majority of patients found the information packages easy to understand (88%) and useful (89%), and no differences were detected between packages. The cost of production and dissemination of NPIP was more than double the cost for mini-NPIP: $8.93 *vs* $3.98 (Canadian dollars) per patient. For patients presenting to a cancer centre for the first time, packages of procedural information do not appear to reduce psychological distress, but are preferred by patients. Given the cost of producing NPIP, mini-NPIP is the preferred approach.

**Keywords:** randomised controlled trial; patient information; neoplasm; psychological distress

Supportive cancer care has developed from the premise of providing comprehensive care and includes a wide variety of services offered to cancer patients and their families to help them cope with their cancer experience (Addington-Hall *et al.*, 1993; Coluzzi *et al.*, 1995). Supportive care services consist not only of interventions that support the patient's anti-cancer therapies, such as anti-emetics and bone marrow stimulating factors, but includes pain management, nutritional support, psychological counselling and methods to enhance health provider and patient communication (Smith, 1990; Levy, 1994).

Cancer care is provided along a continuum that starts with the preclinical phase and continues through the diagnostic, treatment, follow-up and cure or terminal phases (Levine, 1995). At each point along the continuum, the patient and his/her family will experience different needs. Previous supportive cancer care research has tended to focus on the treatment or palliative phases of care, but other points along the continuum are just as relevant and require research.

Receiving a diagnosis of cancer presents a formidable problem and challenge for most individuals and their families (Fallowfield, 1988; Holland, 1989). This diagnosis carries considerable stigma and a high degree of uncertainty. In Ontario, patients are often referred to a tertiary cancer centre following diagnosis where their care is provided by an oncologist and a team of health professionals, e.g. primary care nurse, nutritionist, etc. Thus, when confronted with a new and threatening diagnosis, patients and their families are also faced with the unfamiliarity of the cancer care delivery system. Clinicians at our centre were concerned about patients' emotional and informational needs following their diagnosis and before being seen at the cancer centre. Patients may have two types of informational needs at diagnosis before referral to a regional cancer centre. The first is for specific information related to their cancer, e.g. prognosis and details of treatment. The second relates to procedural information on the processes of care. This type of practical information includes information about resources available to patients, such as treatment services, emotional support programmes, nutritional information, etc. Before the first visit, uncertainties caused by lack of disease-specific information are unavoidable whereas uncertainty owing to the lack of information on procedural matters can potentially be minimised.

The need for information is one of the most frequently cited self-perceived needs identified by cancer patients and their families (Mor *et al.*, 1987; Houts *et al.*, 1988; Canadian Cancer Society, 1992). Randomised trials have demonstrated that treatment- and disease-specific information provided to patients during the course of treatment or in the advanced/terminal stages of illness increases patient knowledge, and several trials have shown an increase in patient satisfaction and a decrease in emotional distress (Morrow *et al.*, 1978; Dodd and Mood, 1981; Dodd, 1982; Israel and Mood, 1982; Johnson, 1982; Rainey, 1985; Simes *et al.*, 1986; Rimer *et al.*, 1987; Johnson, 1988; Dodd, 1988; Derdiarian, 1989; Damian and Tattersall, 1991; Pruitt *et al.*, 1992; North *et al.*, 1992; Dunn *et al.*, 1993). However, limited data are available

Correspondence: T Whelan, Supportive Cancer Care Research Unit, c/o OCTRF Hamilton Regional Cancer Centre, 699 Concession Street, Level 3-60, Hamilton, Ontario L8V 5C2, Canada
Received 22 September 1995; revised 3 January 1996; accepted 8 January 1996

regarding the effect of providing procedural information to newly diagnosed patients with cancer at the point of entry into the cancer-specific health care system.

As part of our programme in supportive cancer care research, we wanted to develop and evaluate an information package that would provide newly diagnosed cancer patients attending a regional cancer centre with information regarding the cancer centre and the process of delivery of care. The information package would be mailed to patients before their first appointment at a regional cancer centre. A randomised trial was designed to assess the extent to which the new patient information package or a mini-version of the same package reduces the psychological distress and meets the information needs of these patients.

### Methods

### *Development of patient information packages*

Before the study, the cancer centre did not provide patients with information in a formal fashion before their initial appointment, nor was there a systematic approach to the provision of information at the time of their initial appointment. A cross-sectional survey of new patients with a variety of primary cancers referred to the Ontario Cancer Treatment and Research Foundation Hamilton Regional Cancer Centre (HRCC) had described the information desired by oncology patients before their initial appointment. A new patient information package (NPIP) and a mini new patient information package (mini-NPIP) were developed on the basis of the results of this survey and information needs identified by representatives of the centre's health care teams and administrative staff, including patient services personnel from the Canadian Cancer Society.

The point of entry into the cancer-specific health care system represents a time when distress and uncertainty for patients may be high. The purpose of the information packages was to provide patients and their families with practical information regarding services provided at the cancer centre and processes of care, e.g. 'what to expect at the first visit'. This was general information and was not disease site or treatment specific. The educational literature suggested that in situations where emotional distress may be experienced, information should be provided using brief, easy to read written materials (Meade *et al.*, 1992; Tackett, 1992). The comprehensive version of the new patient information package (NPIP) had its content format on ten sheets of paper organised in a step-wise format in a folder. This permitted patients and their family members to scan and select information easily from a menu of topics including the cancer centre location, a description of the health care team, treatment services, research and educational activities at the centre and accommodation and community services provided. The mini-version (mini-NPIP) was a condensed version of the information contained in the NPIP. The information topics selected for this package included information about what to expect at the first visit, directions to the centre, a map and parking information. In addition to the patient information, both versions of the information package consisted of: (1) a personalised letter of introduction meant to convey commitment on the part of the HRCC to individual patient care; (2) the name and telephone number of a contact person at the centre who might provide additional information; and (3) a question/answer sheet for the patient to assist in organising questions to be addressed to the health care team and to act as an aid to memory at the initial appointment.

Centre representatives examined the NPIP and mini-NPIP for accuracy, scope of coverage, format and readability. Both versions were piloted with patients in a feasibility study.

### *Feasibility study*

The feasibility study was conducted to examine the clarity, comprehensiveness and acceptability of the NPIP and mini-NPIP from the persepective of the patient and their relatives; to develop and test the feasibility of procedures for distributing the information packages; and to develop operational procedures for the major study of effectiveness and efficiency.

The pretest consisted of a convenience sample of 45 newly referred patients who represented a variety of disease sites and ages. Confirmation that the patient was aware of the appointment was obtained from the referring physician's office. Subjects in the NPIP ($n=17$) and mini-NPIP ($n=16$) groups were selected from lists of newly referred patients according to the date of appointment. The control group ($n=12$) subjects did not receive any information which was the usual practice. The latter group was recruited by requesting the participation of patients who arrived 30 min or more before their first clinic appointment. The NPIP or mini-NPIP was mailed to a subject's home before their initial appointment. Each subject was interviewed on arrival at the cancer centre just before their first clinic appointment regarding the information package's clarity, comprehensiveness, usefulness and any other concerns or questions that had arisen. Patients were also administered the Brief Symptom Inventory (BSI), the shortened version of the Symptom Distress Check List-90 (SCL-90-R) as a global measure of psychological distress (Derogatis, 1982, 1992).

Most patients and relatives reported the information was easy to understand. The map indicating the location of the cancer centre was found to be confusing by some patients and modifications were subsequently made. The need for additional general, not cancer-specific, information was not identified by patients and relatives. Overall, both versions of the information package were viewed as useful by most patients and relatives. Patients indicated that receiving information before their appointment permitted them to be better prepared and to involve the family when desired. A number of patients stated that the package conveyed a message of caring and concern by the cancer centre. Only one patient who had been previously informed of her diagnosis reported being upset by the information.

With respect to operational issues, the process of identifying new patients from referral forms on a daily basis appeared quite feasible. When the interval between the referral request and appointment was less than 7 days, it was not practical to mail out information. Patients also indicated that they would prefer confidentiality for mailed information so that the envelope containing the information should have no identification that it was sent from the cancer centre.

On average, 20 min were needed to conduct the patient interview including the BSI. The interviews occurred in several disease site clinics without disruption to the clinic schedule, and these disease site clinics were chosen for the major study.

### *Randomised trial design*

We studied consecutive patients with newly diagnosed breast, gynaecological, lung and prostate cancer attending the cancer centre for the first time. New patients were identified on a daily basis through the referral forms to the cancer centre. For a patient to be eligible for inclusion to the study, their appointment had to be confirmed and they had to have a valid mailing address.

Eligible patients were prerandomised to one of three interventions: (1) new patient information package (NPIP), (2) mini version of the new patient information package (mini-NPIP) or (3) no information package. Stratification occurred according to disease site: breast, gynaecological, lung or prostate. Patients randomised to NPIP or mini-NPIP were mailed the information package at least one week before their first appointment, but were not introduced to the concept of the study at this time. Patients in the control group were not mailed any information.

On arrival at the cancer centre, approximately 30 min before their appointment, patients and relatives were approached regarding the study and consent to be interviewed. Patients and their relatives in the following categories were not interviewed and were considered post-randomisation exclusions: (1) patient too ill to complete the interview; (2) non-English speaking; (3) arrived too late for interview; (4) previous diagnosis of cancer; (5) appointment cancelled owing to other administrative reasons; (6) failure to give informed consent.

All interviews were conducted by a skilled research assistant. Patients were interviewed regarding background demographic variables, level of activity and level of pain. They were then administered the BSI (Derogatis, 1982, 1992; Derogatis and Melisaratos, 1983) and the Sherer Self-Efficacy Scale (Sherer and Maddux, 1982; Sherer and Adams, 1983). The BSI provides a global measure of psychological distress 'in the past seven days including today' and has nine subscales including anxiety and depression. This self-administered scale has proven psychometric properties (Derogatis, 1982, 1992; Derogatis and Melisaratos, 1983) and has been used extensively in cancer patients (Pruitt *et al.*, 1992; Stefanek *et al.*, 1987; Edwards *et al.*, 1985; Schain *et al.*, 1985; Wellisch *et al.*, 1991; Schover *et al.*, 1989). Each of the 53 items are scored 0–4 (0, not at all distressed and 4, extremely distressed) yielding a summary score between 0 and 212. The General Severity Index (GSI) is the most sensitive indicator of emotional distress level combining data on a number of symptoms and intensity of distress. A GSI score is calculated by dividing the subject's total score by the number of items ($n=53$). This raw score is then converted to a standardised T-score. The Sherer Self-Efficacy Scale is a 30-item scale that measures expectations of self-efficacy that are not tied to specific situations (data not shown).

Following administration of these scales, the patients were administered a questionnaire consisting of items developed during the feasibility study. Items included a patient's expectations of care and fear regarding the initial appointment; preferences for information in general and by which method; understanding and usefulness of the information package sent; and usefulness of the question/answer sheet. Understanding and usefulness were assessed using a five-point Likert scale response option (e.g. 1, extremely easy to understand and 5, very difficult to understand; and 1, very useful and 5, not at all useful). During administration of the patient questionnaire, relatives of patients who had received an information package were interviewed regarding whether they had read the information package, their understanding and the usefulness of the information package sent. Patients and relatives in the control group who were not mailed any information were not asked specific questions regarding the information packages, but were given the comprehensive package following the interview.

### *Statistical analysis*

Mean BSI scores for each group, as measured by the GSI, and for each subscale were compared by one-way ANOVA. Linear regression models were used to adjust comparisons for any imbalance in baseline characteristics. Other outcomes, e.g. patient preferences, understanding and usefulness of the information packages expressed as frequencies, were compared using chi-square contingency table lists.

### *Economic evaluation*

The cost of producing and using NPIP and mini-NPIP was determined taking into account the administrative, printing and postage costs. Unit prices obtained for this study were in 1992 Canadian dollars (in 1992, $1.00 Canadian was equivalent to approximately £2.10). The primary viewpoint of the analysis was that of costs borne by the cancer centre.

### Results

### *Patient population*

A total of 465 patients were randomised. There were 53 patients excluded post-randomisation in the NPIP group, 46 in the mini-NPIP group and 62 in the control group. Thus 100 patients in the NPIP group, 102 in the mini-NPIP group and 102 patients in the control group completed the interviews and contributed data to the analysis. The reasons for exclusion by study group are listed in Table I and were primarily for administrative reasons where the appointment time had been changed, or for a previous diagnosis of cancer. The reasons for exclusion were similar between groups, although the patients from the no information control group were more often excluded because they arrived late and were not available for interview. Patients excluded were similar in age but were more often male.

The treatment groups were comparable in terms of age, sex, employment and marital status, disease site and level of activity (see Table II). Patients in the mini-NPIP group were observed to be more often college educated. Patients in the NPIP group were observed to experience more pain.

### *Psychological distress*

Six patients in the NPIP group and four in the mini-NPIP group had not received the information packages in the mail. These patients were included in the analysis. The patient mean scores for psychological distress as measured by the GSI or for any of the subscales did not differ between groups (data for the GSI, anxiety and depression subscales shown in Table III). The GSI and anxiety subscale were affected by whether or not a patient experienced pain. When this was adjusted for in the regression analysis, the results did not change.

### *Patient preferences and expectations*

Patients were asked whether they preferred to receive information from the centre, at what time and by what means. Overall 98% of patients preferred to receive information and there was no difference between groups. Over 83% of patients preferred to receive information before the first appointment, 6% after the first visit, 4% upon arrival and 4% had no preference. Patients who had received the information packages were more likely to prefer to receive the information before they arrived (94% *vs* 62%, $P<0.001$). Patients were also asked the method by which they preferred to receive the information: 79% preferred to receive the information by mail, 7% by telephone contact, 5% by pamphlet available at the doctor's office, 1% as a prerecorded telephone message and 8% of patients had no preference or expressed an alternative source. Again, patients who had received the information packages were more likely to prefer receiving information by mail than those who did not (84% *vs* 69%, $P=0.001$). Overall, 49% of patients expressed dread or fear regarding their initial appointment

**Table I** Reason for exclusion by study group

| | *NPIP* | *Mini-NPIP* | *Control* |
|---|---|---|---|
| Too ill or deceased | 6 | 7 | 4 |
| Non-English speaking | 4 | 3 | 1 |
| Previous diagnosis of cancer | 16 | 11 | 6 |
| Appointment changed or cancelled | 14 | 12 | 16 |
| Arrived too late | 9 | 11 | 30* |
| Did not consent | 1 | 0 | 2 |
| Other | 3 | 2 | 3 |
| Total exclusions | 53 | 46 | 62 |

*$P=0.001$ ($\chi^2$, three-way comparison).

Table II Baseline characteristics by study group

| | NPIP (n=100) | Mini-NPIP (n=102) | Control (n=102) |
|---|---|---|---|
| Age (mean) | 64 | 61 | 64 |
| Sex (female) | 58 | 60 | 61 |
| Education | | | |
| ≤Primary | 25 | 15 | 19 |
| Secondary | 56 | 49 | 58 |
| College or university | 18 | 36 | 24 |
| Employement | | | |
| Full-time | 11 | 16 | 11 |
| Part-time or homemaker | 16 | 21 | 18 |
| Retired | 58 | 51 | 52 |
| Unemployed sick leave | 13 | 12 | 21 |
| Marital status | | | |
| Cohabiting or married | 68 | 81 | 75 |
| Single, separated or divorced | 10 | 7 | 12 |
| Widowed | 20 | 10 | 10 |
| Disease site | | | |
| Breast | 25 | 25 | 25 |
| Gynaecological | 25 | 26 | 27 |
| Lung | 25 | 26 | 25 |
| Prostate | 25 | 25 | 25 |
| Activity level | | | |
| Fully active | 45 | 57 | 48 |
| Restricted in some activity | 36 | 30 | 37 |
| Unable to work | 13 | 12 | 12 |
| Confined to bed≥50% | 4 | 3 | 5 |
| Experiencing pain | 40 | 30 | 33 |

Table III Mean (s.d.) BSI T-scores by study group*

| | NPIP | | Mini-NPIP | | Control | |
|---|---|---|---|---|---|---|
| Depression dimension | 52.6 | (9.1) | 53.0 | (9.3) | 53.6 | (9.8) |
| Anxiety dimension | 54.7 | (10.2) | 54.9 | (10.7) | 54.6 | (10.8) |
| General Severity Index | 53.8 | (8.3) | 53.6 | (10.1) | 53.9 | (10.1) |

*$P$ >0.5 for all comparisons.

and 89% expected to receive good quality care at the centre. No difference was demonstrated between groups.

*Acceptibility of information packages*

Eleven patients in the NPIP group and three patients in the mini-NPIP group did not read the information packages ($P=0.02$). Overall, 88% of patients found the information packages easy to understand and a greater percentage of patients found the mini-NPIP extremely easy to understand (73% *vs* 55%, $P=0.01$). A total of 89% of patients found the information packages useful and again a trend was noticed where a greater percentage of patients found the mini-NPIP very useful (61% *vs* 49%, $P=0.06$). Patient understanding and usefulness was affected by level of education, and when this was adjusted for by logistic regression, the differences between information packages were no longer evident. All topics within the respective packages were found to be useful ranging from 72% of patients reporting information regarding the administrative structure of the clinic as useful to 88% reporting information concerning what to expect at their first visit as being useful. In all 50% of patients used the question answer sheet and there was no difference between information packages.

*Relative interview*

Some 69% of patients were accompanied by a relative who was female in 49% of these cases. Of relatives attending the centre 76% had read the information package and there was no difference between packages. The majority of relatives who read the information reported it easy to understand (96%) and useful (98%). Again, there was no difference between information packages.

*Economic evaluation*

For the purposes of this evaluation, the effectiveness of the two information packages was assumed to be equivalent and a cost minimisation analysis was performed. The cost of identifying patients, printing information and mailing was determined in Canadian dollars per patient for each information package (Table IV). The cost of production and use of NPIP was more than double the cost for mini-NPIP. Assuming our centre receives approximately 5000 new patients per year, the use of a mini-NPIP would result in savings of $24 750 per year.

### Discussion

The provision of health-related information to patients with cancer may have many effects: increasing knowledge; increasing satisfaction; enhancing self-care and compliance leading to better health outcomes; increasing involvement in decision-making; and reducing anxiety and distress (Fernsler and Cannon, 1991).

A review of the literature from 1980 to the present identified 15 randomised trials evaluating interventions to provide information to cancer patients (Morrow *et al.*, 1978; Dodd and Mood, 1981; Dodd, 1982; Israel and Mood, 1982; Johnson, 1982; Rainey, 1985; Simes *et al.*, 1986; Rimer *et al.*, 1987; Johnson, 1988; Dodd, 1988; Derdiarian, 1989; Damian and Tattersall, 1991; Pruitt *et al.*, 1992; North *et al.*, 1992; Dunn *et al.*, 1993).

Acknowledging that the sample size was small in most studies ($n<100$ in 13 of the 15 studies identified), many reported positive results: nine out of 12 reported an increase in patient knowledge and recall, and three reported a trend in this direction; four out of six reported a decrease in emotional distress, anxiety or depression, and one reported a trend in this direction; three out of four reported an increase in patient satisfaction and one reported a trend in this direction; and two out of two studies reported an increase in patient compliance or change in behaviour as a result of the information intervention. Recognising the positive results, all of these studies have limited their evaluations to the provision of disease-specific information to cancer patients during the treatment or follow-up phase of their illness. We identified only two studies that identified patients immediately after their first consultation with their oncologist (Derdiarian, 1989; Dunn *et al.*, 1993) and we were unable to identify any study that evaluated the provision of procedural information before this.

We were interested in studying how the provision of information about services available to patients and the processes of care before their arrival at a regional cancer

Table IV Economic considerations: cost ($ Canadian) of NPIP *vs* mini-NPIP per patient

| | NPIP | Mini-NPIP |
|---|---|---|
| Printing | 5.00 | 1.00 |
| Folder | 0.50 | 0.00 |
| Envelope | 0.07 | 0.05 |
| Postage | 0.86 | 0.43 |
| Secretarial time[a] | 2.50 | 2.50 |
| Total | 8.93 | 3.98 |
| Cost to cancer centre per year[b] | $44 650 | $19 900 |

[a] Equivalent 10 min per patient at $15.00 per hour for time spent to retrieve names and addresses of patients, organising informational material and typing letters and labels. [b] Assume 5000 new patients seen per year.

centre might affect newly diagnosed patients with cancer. Receiving a diagnosis of cancer is a catastrophic event for most individuals and their families and is accompanied by a high degree of uncertainty. When people do not know what to expect and the degree of uncertainty is high, the subjective experience is often one of anxiety or emotional distress. The provision of information may reduce anxiety or distress. Our results suggest that while the provision of procedural information to patients did not substantially affect their level of psychological distress, the vast majority would prefer to receive it. This was more evident for patients who received the information packages suggesting that the information itself was beneficial. Although we did not formally test for knowledge or recall, patients frequently reported that the information provided made them feel more prepared for their visit to the cancer centre.

The observation that the provision of information did not increase or decrease patient distress is important, both for the lack of a negative or a positive effect. Simes *et al.* (1986) reported a randomised trial that compared the provision of detailed information and a consent form to cancer patients about to undergo therapy with an individual approach at the physician's discretion. They observed increased anxiety in patients who had received the detailed information with this effect dissipating over time. Similarly, we were concerned that for some patients the new patient information package might be upsetting by reminding them of their recent diagnosis and impending visit to the cancer centre. During the feasibility study, several patients had asked that the information from the centre be sent confidentially with no identification of the sender. One patient described the information as quite upsetting. We were pleased to find that, on average, the information package did not increase patient distress.

As indicated earlier, most studies that have evaluated the provision of information on emotional distress have shown a reduction in anxiety or depression. The fact that the provision of information was not shown to reduce the psychological distress in this study may have occurred as a result of several different factors. The information presented was procedural and general in context, rather than specific information for patients regarding their disease, prognosis and available treatments. Derdiarian (1989) found that patients newly diagnosed with cancer attached the highest importance to information pertaining to their disease and its consequences and less importance to that of a social or practical nature. Thus, it may be that although the type of information we presented is useful to patients as documented in the study, it does not have a marked effect on psychological distress. Another possibility, however, is that the information truly did reduce distress to some modest degree, but attending the cancer centre for the first time was relatively stressful and any effect of the information package was not evident at this time. Finally, the Brief Symptom Inventory was initially developed to discriminate major psychological morbidity (Derogatis, 1982, 1992; Derogatis and Melisaratos, 1983). We chose to use this instrument as our outcome measure because it had been used extensively in oncology studies, it provided a global measure of distress, and a previous trial in cancer patients had shown it to be responsive to an information intervention (Pruitt *et al.*, 1992). However, it still may be that this instrument was not sufficiently sensitive to detect clinically important differences in psychological distress between groups. A more specific instrument measuring anxiety, e.g. Spielberger State–Trait Anxiety Inventory may have detected a more subtle difference between the groups (Spielberger *et al.*, 1970).

A potential limitation of our study is that it employed a prerandomisation design with post-randomisation exclusions. Although exclusions were reasonably balanced between the treatment groups, the groups did differ in terms of the number of patients excluded because of an increased number of late arrivals in the control group. Such an effect could have resulted in a more highly selected control sample. When we reviewed our data, late arrivals were more evenly distributed between the study groups in the breast and gynaecology clinics. There was no interaction between treatment effect and disease site (breast and gynaecology *vs* others) suggesting that the increased number of late exclusions in the control group did not bias our results.

With respect to the information packages themselves, our results suggest that the smaller, less glossy version was as useful to patients as the larger, more comprehensive package suggesting for this type of information that the smaller package was sufficient. A cost minimisation analysis indicated that the adoption of a policy of using the former package rather than the latter could save approximately $5 (Canadian) per patient or $25 000 per year for our centre. When one considers that some centres in Ontario are providing even more expensive information packages, the possible savings may be greater. In times of fiscal constraint and potential rationing of health care, these monies might be directed to better use.

This study underlines some of the problems and difficulties in measuring the effect of the provision of procedural information to patients before their attendance at a cancer clinic. We were unable to show an effect of such information on patients' level of psychological distress, but the majority of our patients preferred to receive this type of information. Assessment of the effect of the information on patients' knowledge, or behaviour in terms of accessing other information sources may have provided additional support for this preference. Based on the results of this study, we are currently providing our patients with the mini-version of the New Patient Information Package before their first appointment at the cancer centre.

**Acknowledgement**

This work was supported by the Ontario Ministry of Health, Health System-linked Research Programme.

### References

ADDINGTON-HALL JM, WEIR MW, ZOLLMAN C AND McILLMURRAY MB. (1993). A national survey of the provision of support services for people with cancer. *Br. Med. J.*, **306**, 1649–1650.

CANADIAN CANCER SOCIETY. (1992). *The Final Report on the Needs of Persons Living with Cancer Across Canada.* Canadian Cancer Society: Toronto.

COLUZZI PH, GRANT M, DOROSHOW JH, RHINER M, FERRELL B AND RIVERA L. (1995). Survey of the provision of supportive care services at National Cancer Institute-designated cancer centres. *J. Clin. Oncol.*, **13**, 756–764.

DAMIAN D AND TATTERSALL MHN. (1991). Letters to patients: Improving communication in cancer care. *Lancet*, **338**, 923–925.

DERDIARIAN AK. (1989). Effects of information on recently diagnosed cancer patients' and spouses' satisfaction with care. *Cancer Nurs.*, **12**, 285–292.

DEROGATIS LR. (1982, 1992). *The Brief Symptom Inventory (BSI). Administration, Scoring & Procedures Manual - II.* Clinical Psychometric Research: Baltimore.

DEROGATIS LR AND MELISARATOS N. (1983). The Brief Symptom Inventory (BSI): an introductory report. *Pyschol. Med.*, **13**, 595–605.

DODD MJ. (1982). Cancer patients' knowledge of chemotherapy: assessment and informational interventions. *Oncol. Nurs. Forum*, **9**, 39–44.

DODD MJ. (1988). Efficacy of proactive information on self-care in chemotherapy patients. *Patient Educ. Couns.*, **11**, 215–225.

DODD MJ AND MOOD DW. (1981). Chemotherapy: helping patients to know the drugs they are receiving and their possible side-effects. *Cancer Nurs.*, **4**, 311–318.

DUNN SM, BUTOW PN, TATTERSALL MHN, JONES QJ, SHELDON JS, TAYLOR JJ AND SUMICH MD. (1993). General information tapes inhibit recall of the cancer consultation. *J. Clin. Oncol.*, **11**, 2279–2285.

EDWARDS J, DICLEMENTE C AND SAMUELS ML. (1985). Psychological characteristics: a pretreatment survival marker of patients with testicular cancer. *J. Psychosoc. Oncol.*, **3**, 79–94.

FALLOWFIELD LJ. (1988). The psychological complications of malignant disease. In *Medical Complications of Malignant Disease*, Kaye S-B and Rankin EM (eds). *Bailleres Clin. Oncol.*, **2**, 461–478.

FERNSLER JI AND CANNON CA. (1991). The whys of patient education. *Semin. Oncol. Nurs.*, **7**, 79–86.

HOLLAND JC. (1989). Clinical course of cancer. In *Handbook of Psycho-Oncology*, Holland JC and Rowland J (eds). pp. 75–100. Oxford University Press: New York, NY.

HOUTS PS, YASKO JM, HARVEY HA, KAHN SB, HARTZ AJ, HERMANN JF, SCHELZEL GW AND BARTHOLOMEW MJ. (1988). Unmet needs of persons with cancer in Pennsylvania during the period of terminal care. *Cancer*, **62**, 627–634.

ISRAEL MJ AND MOOD DW. (1982). Three media presentations for patients receiving radiation therapy. *Cancer Nurs.*, **5**, 57–63.

JOHNSON J. (1982). The effects of a patient education course on persons with a chronic illness. *Cancer Nurs.*, **5**, 117–123.

JOHNSON JE. (1988). Reducing the negative impact of radiation therapy on functional status. *Cancer*, **61**, 46–51.

LEVINE MN. (1995). The clinical problem: differing expectations and outcomes from the perspectives of the patient and the oncologist. In *The Challenges in Studying the Socioeconomic Dimensions in Cancer Therapy*, Champey Y and Hillman AL (eds). pp. 23–28. Proceedings of a Symposium held in Philadelphia, PA, USA, November 1994, The Medicine Group USA, Inc.: Yardley, PA.

LEVY MH. (1994). Supportive Oncology. *Semin. Oncol.*, **21**, 699–700.

MEADE CD, DIEKMANN J AND THORNHILL DG. (1992). Readability of American Cancer Society patient education literature. *Oncol. Nurs. Forum*, **19**, 51–55.

MOR V, GUADAGNOLI E AND WOOL M. (1987). An examination of the concrete service needs of advanced cancer patients. *J. Psychosoc. Oncol.*, **5**, 1–17.

MORROW G, GOOTNICK J AND SCHMALE A. (1978). A simple technique for increasing cancer patients' knowledge of informed consent to treatment. *Cancer*, **42**, 793–799.

NORTH N, CORNBLEERT MA, KNOWLES G AND LEONARD RCF. (1992). Information giving in oncology: a preliminary study of tape-recorder use. *Br. J. Clin. Psychol.*, **31**, 357–359.

PRUITT BT, WALIGORA-SERAFIN B, MCMAHON T, BYRD G, BESSLEMAN L, KELLY GM, DRAKE DA AND CUELLAR D. (1992). Relief of psychological distress in oncology outpatients by an education intervention (abstract 1312). *Proc. Am. Soc. Clin. Oncol.*, **11**, 379.

RAINEY LC. (1985). Effects of preparatory patient education for radiation oncology patients. *Cancer*, **56**, 1056–1061.

RIMER B, LEVY MH, KEINTZ ME, FOX L, ENGSTROM PF AND MACELWEE N. (1987). Enhancing cancer pain control regimes through patient education. *Patient Educ. Couns.*, **10**, 267–277.

SCHAIN WS, WELLISCH DK, PASNAU RO AND LANDSVERK J. (1985). The sooner the better: a study of psychological factors in women undergoing immediate versus delayed breast reconstruction. *Am. J. Psychiatry*, **142**, 40–46.

SCHOVER LR, FIFE M AND GERSHENSON DM. (1989). Sexual dysfunction and treatment for early stage cervical cancer. *Cancer*, **63**, 202–212.

SHERER M AND ADAMS CH. (1983). Construct validation of the self-efficacy scale. *Psychol. Rep.*, **53**, 899–902.

SHERER M AND MADDUX JE. (1982). The self-efficacy scale: construction and validation. *Psychol. Rep.*, **51**, 663–671.

SIMES RJ, TATTERSALL MHN, COATES AS, RAGHAVAN D, SOLOMON HJ AND SMARTT H. (1986). Randomized comparison of procedures for obtaining informed consent in clinical trials of treatment for cancer. *Br. Med. J.*, **293**, 1065–1068.

SMITH T. (1990). Cancer services. Patients with cancer need compassionate care from the start of their illness. *Br. Med. J.*, **301**, 1406–1407.

SPIELBERGER CD, GORSUCH RL AND LUSHENE RE. (1970). *STAI Manual for the State–Trait Anxiety Inventory*. Consulting Psychologists Press, Inc: Palo Alto, CA.

STEFANEK ME, DEROGATIS LR AND SHAW A. (1987). Psychological distress among oncology outpatients: prevalence and severity as measured with the Brief Symptom Inventory. *Psychosomatics*, **28**, 530–539.

TACKETT SS. (1992). Patient education materials: are they readable? *Oncol. Nurs. Forum*, **19**, 83–85.

WELLISCH DK, GRITZ ER, SCHAIN W, WANG JH AND SIAU J. (1991). Psychosocial functioning of daughters of breast cancer patients: daughters and comparison subjects. *Psychosomatics*, **32**, 324–336.